# HDAC inhibitors cause site-specific chromatin remodeling at PU.1-bound enhancers in K562 cells

**DOI:** 10.1186/s13072-016-0065-5

**Published:** 2016-04-16

**Authors:** Christopher L. Frank, Dinesh Manandhar, Raluca Gordân, Gregory E. Crawford

**Affiliations:** Department of Molecular Genetics and Microbiology, Duke University, Durham, NC 27708 USA; Center for Genomic and Computational Biology, Duke University, Durham, NC 27708 USA; Program in Computational Biology and Bioinformatics, Duke University, Durham, NC 27708 USA; Department of Biostatistics and Bioinformatics, Duke University, Durham, NC 27708 USA; Division of Medical Genetics, Department of Pediatrics, Duke University, Durham, NC 27708 USA

**Keywords:** Histone deacetylase inhibitor, Chromatin accessibility, Acetylation, Pioneer factor, Enhancer

## Abstract

**Background:**

Small molecule inhibitors of histone deacetylases (HDACi) hold promise as anticancer agents for particular malignancies. However, clinical use is often confounded by toxicity, perhaps due to indiscriminate hyperacetylation of cellular proteins. Therefore, elucidating the mechanisms by which HDACi trigger differentiation, cell cycle arrest, or apoptosis of cancer cells could inform development of more targeted therapies. We used the myelogenous leukemia line K562 as a model of HDACi-induced differentiation to investigate chromatin accessibility (DNase-seq) and expression (RNA-seq) changes associated with this process.

**Results:**

We identified several thousand specific regulatory elements [~10 % of total DNase I-hypersensitive (DHS) sites] that become significantly more or less accessible with sodium butyrate or suberanilohydroxamic acid treatment. Most of the differential DHS sites display hallmarks of enhancers, including being enriched for non-promoter regions, associating with nearby gene expression changes, and increasing luciferase reporter expression in K562 cells. Differential DHS sites were enriched for key hematopoietic lineage transcription factor motifs, including SPI1 (PU.1), a known pioneer factor. We found PU.1 increases binding at opened DHS sites with HDACi treatment by ChIP-seq, but PU.1 knockdown by shRNA fails to block the chromatin accessibility and expression changes. A machine-learning approach indicates H3K27me3 initially marks PU.1-bound sites that open with HDACi treatment, suggesting these sites are epigenetically poised.

**Conclusions:**

We find HDACi treatment of K562 cells results in site-specific chromatin remodeling at epigenetically poised regulatory elements. PU.1 shows evidence of a pioneer role in this process by marking poised enhancers but is not required for transcriptional activation.

**Electronic supplementary material:**

The online version of this article (doi:10.1186/s13072-016-0065-5) contains supplementary material, which is available to authorized users.

## Background

Large cancer genotyping efforts such as The Cancer Genome Atlas (TCGA) have found epigenetic regulators are frequent targets of oncogenic mutation and translocation. For instance, 76 % of urothelial carcinomas analyzed by TCGA were found to carry at least one inactivating mutation in a chromatin regulatory gene [1]. These types of mutations are thought to lead to aberrant epigenetic states at many loci in the genome that together contribute to expression patterns and signaling cascades that facilitate oncogenic phenotypes. Alongside these discoveries, chromatin regulator-targeted pharmacological therapies have shown the potential to reverse aberrant epigenetic states driving cancer cell proliferation [2]. Histone deacetylase inhibitors (HDACi) represent one class of compounds with this potential.

The majority of HDACi investigated for anticancer application act to block removal of acetyl groups from protein lysines by inhibiting the active sites of zinc-dependent class I, II, and IV histone deacetylases [3, 4]. HDACi are capable of inducing cell cycle arrest, differentiation, and apoptosis in a variety of preclinical cancer models, and thus far three HDACi have been FDA cleared for anticancer applications [3, 4]. In clinical trials, multiple HDACi have been shown to have severe dose-limiting toxicities [5], indicating that a more nuanced understanding of HDACi anticancer effects could be beneficial to the development of more specific and well-tolerated therapies.

Exposure to HDACi is thought to increase chromatin acetylation levels genome wide [6, 7] but in many cases the exact mechanisms by which HDACi stop cancer cell proliferation remain poorly defined. Increased histone tail acetylation levels have classically been associated with relaxation of local chromatin and greater accessibility for transcription factor binding [3, 8]; however, more recent studies show that specific histone acetylation marks demarcate different gene regulatory element functions and are dynamically maintained and read by trans-acting “writer,” “eraser,” and “reader” factors [9, 10]. Furthermore, previous studies report only a fraction (~10 %) of genes respond to HDACi treatment with expression changes, indicating these compounds are more specific than would be expected by global histone hyperacetylation [11, 12]. Therefore, a better understanding of how these drugs impact gene expression is necessary.

The ability of HDACi to induce differentiation responses analogous to the highly effective all-*trans* retinoic acid therapy used in acute promyelocytic leukemia [13] is a particularly interesting application of these compounds that could be useful for resensitizing cancer cells to other chemotherapeutics or eliminating cancer stem cells. For over 30 years, it has been noted that HDACi treatment of the myelogenous leukemia line K562 results in differentiation along an erythrocytic lineage [14, 15], providing a well-characterized system for HDACi-initiated differentiation.

To investigate the relationship between chromatin changes and the transcriptional response to HDACi treatment in the context of induced cancer cell differentiation, we measured genome-wide chromatin accessibility (DNase-seq) and expression (RNA-seq) changes resulting from sublethal HDACi treatment of K562 cells. As cell proliferation slowed, we detected several thousand gene regulatory elements where chromatin accessibility increased or decreased. These changes coincide with nearby gene expression changes and likely represent enhancer element activation or deactivation events. Motif enrichment analysis indicated that the pioneer factor PU.1 was bound to many of the newly opened DHS sites, which we confirmed by ChIP-seq. Since PU.1 is known to be involved in hematopoietic cell differentiation [16, 17], we tested whether overexpression and knockdown of PU.1 could explain the HDACi observed changes in chromatin and expression. Overexpression of PU.1 modestly opened the DHS sites shown to be opened by HDACi treatment, and shRNA-mediated knockdown of PU.1 failed to block the chromatin accessibility and gene expression changes associated with HDACi treatment. Together, this suggests that while PU.1 is present at sites of HDACi-induced chromatin changes, this factor is not the primary driver of these changes. Instead, a machine-learning approach suggests that enrichment of H3K27me3 specifically marks HDACi-responsive DHS sites. These findings add to our mechanistic knowledge of how HDACi alter chromatin and gene expression patterns, induce differentiation, and ultimately block cancer cell proliferation.

## Results

### HDACi drive site-specific chromatin accessibility changes in K562 cells

**Fig. 1:**
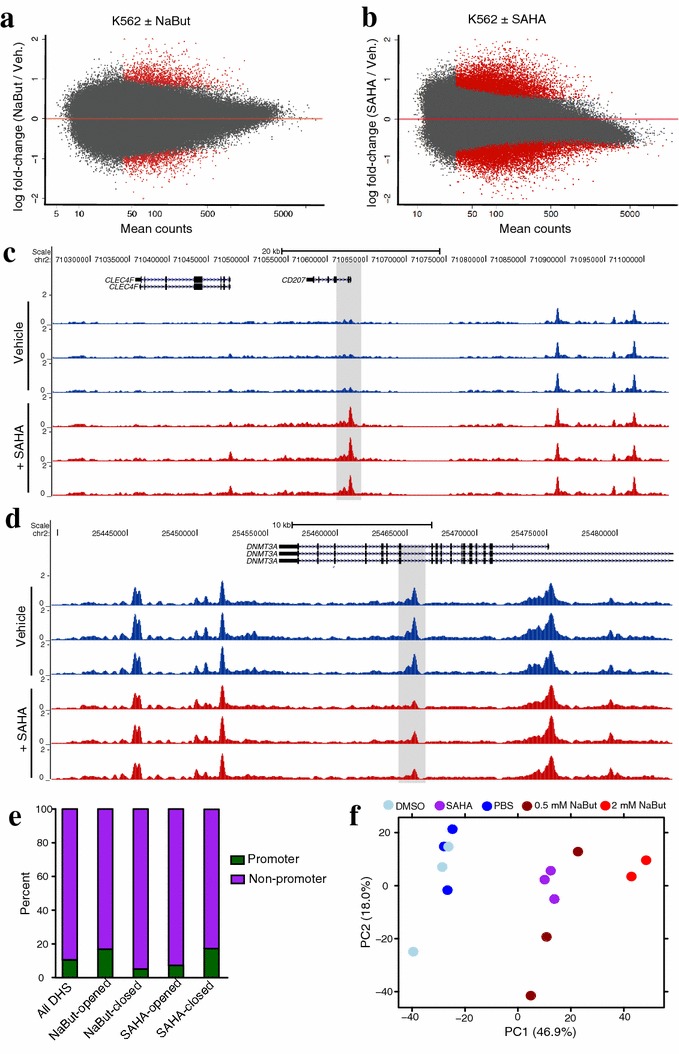
HDACi treatment induces site-specific chromatin remodeling in K562 cells. **a** MA plot of fold-change in K562 chromatin accessibility (DNase-seq signal) over average signal found at each site following 72-h 0.5 mM NaBut treatment. *Red marks* DHS sites with significantly changed chromatin accessibility (FDR < 0.05, *n* = 3 replicates). **b** MA plot of fold-change in K562 chromatin accessibility over average signal following 72-h 1 μM SAHA treatment. *Red marks* DHS sites with significantly changed chromatin accessibility (FDR < 0.05, *n* = 3 replicates). **c** Representative example of SAHA-opened DHS site found near the promoter of *CD207*. **d** Representative example of SAHA-closed DHS site found in an intron of *DNMT3A*. **e** Distribution of all DHS sites and differential DHS sites between promoter (< 2 kb from TSS) and non-promoter location. **f** Regularized log-transformed DNase-seq data for vehicle control K562 (DMSO or PBS) and HDACi-treated K562 (0.5 mM NaBut, 2 mM NaBut, or 1 μM SAHA) plotted in first two principal components of PCA

To assess the extent of chromatin accessibility changes cancer cells might undergo as a result of HDACi treatment, we performed DNase-seq on the myelogenous leukemia line K562 following 72-h incubations with the 0.5 mM sodium butyrate (NaBut) or 1 μM suberanilohydroxamic acid (SAHA). These concentrations were chosen as high enough to slow K562 proliferation by ~50 % but limit cell death at 72 h to less than 10 %. DESeq2 [18] was used to quantitatively compare DNase signal from vehicle control to treated cells (Fig. 1a, b; Additional file 1: Table S1). Our analysis found roughly equal numbers of DHS sites that significantly “open” and “close” in response to treatment: NaBut treatment resulted in 1151 opening DHS sites and 1132 closing DHS sites, while SAHA resulted in 7962 opening DHS sites and 10,349 closing (FDR < 0.05). We note that many of these DHS sites display remarkably specific accessibility changes amongst surrounding DHS sites that do not change (Fig. 1c, d). The majority of the HDACi-opened and HDACi-closed DHS sites are located outside of proximal promoter regions (Fig. 1e), suggesting these elements may be distal enhancers.

Although the two drug treatments resulted in approximately tenfold different totals of differential DHS sites, we found the direction of signal changes was remarkably similar between drugs across these sites (Additional file 2: Fig. S1A–D). Additionally, increasing NaBut concentration fourfold to 2 mM over the same 72-h treatment resulted in a 15,599 additional differential DHS sites at FDR < 0.05 (Additional file 2: Fig. S1E). This suggests the two HDACi are capable of inducing similar chromatin accessibility changes and that the differences in the number of differential DNase sites are likely due to inhibitor potency. Indeed, a principle components analysis separates vehicle controls from both drug-treated populations along the first principal component (Fig. 1f), implying the greatest source of variation in these data is an HDACi response in common to NaBut and SAHA. Clustering samples by Euclidean distance between regularized log-transformed DHS signal (Additional file 2: Fig. S1F) further supports the notion these two drug treatments result in similar global chromatin accessibility changes in K562 cells.

### HDACi-opened and HDACi-closed DHS sites associate with directional gene expression changes

**Fig. 2:**
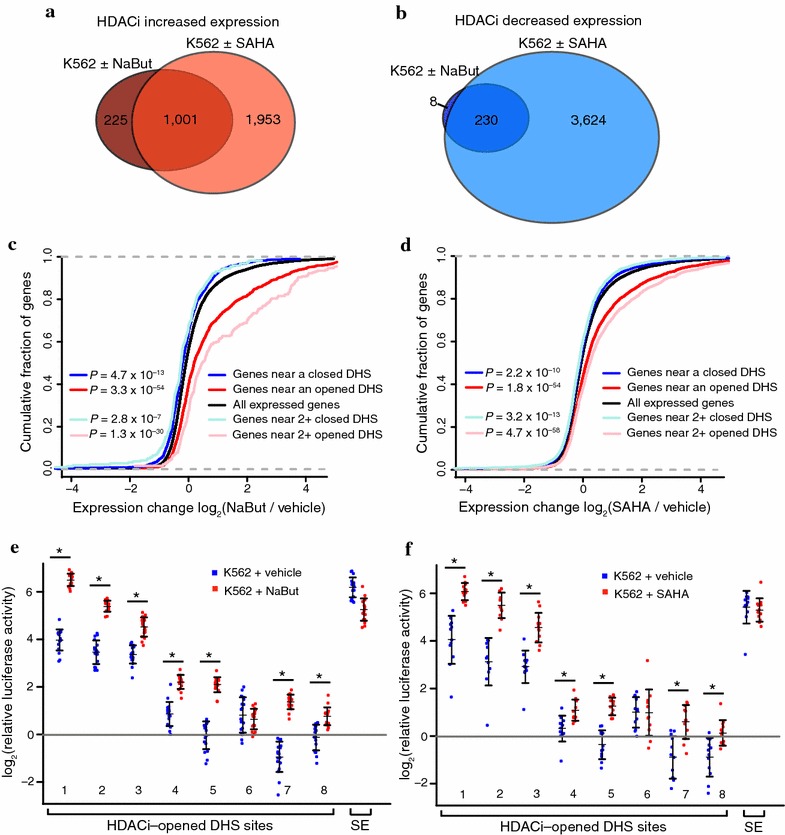
Chromatin accessibility changes are associated with transcriptional changes from HDACi treatment. Overlap between sets of up (**a**)- or down (**b**)-regulated genes (FDR < 0.05) following 72-h NaBut (*n* = 3 replicates) or SAHA treatment (*n* = 2 replicates) of K562 cells. Association of chromatin accessibility changes with nearby changes in gene expression resulting from NaBut (**c**) or SAHA (**d**) treatment. *Plots* show the cumulative fraction of genes exhibiting that level of fold-change in expression on the *x*-axis. Sets of all expressed genes, genes closest to an opened DHS site, genes closest to a closed DHS site, genes closest to 2 or more opened DHS sites, and genes closest to 2 or more closed DHS sites are plotted. Mann–Whitney test used to assess significance between gene sets and all expressed genes. **e**, **f** Luciferase assay results for eight DHS sites cloned in front of a minimal promoter (*pGL4.23*) that open following HDACi treatment, and a known strong enhancer (SE) control. *Data points* show 12–18 replicates per construct with mean and standard deviation in *black*. *Blue* points mark level of normalized luciferase activity in K562 cells with vehicle control (1× PBS or DMSO) added and points in *red* show level of normalized luciferase activity with 1 mM NaBut or 1 μM SAHA added for 24 h. Note *y*-axis is on log scale. *Asterisk* (*) denotes significant by Bonferroni-corrected two-sided *T* test (*P* = 2.8 × 10^−13^, 2.4 × 10^−15^, 3.6 × 10^−7^, 1.6 × 10^−10^, 4.5 × 10^−12^, 0.17, 1.8 × 10^−12^, and 3.5 × 10^−5^ from *left* to *right* for NaBut; *P* = 8.1 × 10^−8^, 5.0 × 10^−6^, 2.2 × 10^−4^, 0.0016, 3.0 × 10^−7^, 0.65, 3.7 × 10^−4^, and 0.0040 from *left* to *right* for SAHA)

RNA-seq was used to investigate the relationship between the HDACi-induced chromatin accessibility changes and transcriptional regulation. Using a FDR of 0.05, we identified 1464 differentially expressed genes with NaBut treatment and 6808 differentially expressed genes in the SAHA treatment (Additional file 3: Table S2). A high degree of overlap was observed between treatments with the two HDACi compounds (Fig. 2a, b). Associating each DHS site with its nearest gene and plotting the cumulative fraction of genes that display an increasing fold-change of expression level from vehicle to HDACi treatment revealed a strong association between locally opening DHS sites and increased gene expression for both HDACi (Mann–Whitney test, *P* = 3.3 × 10^−54^ for NaBut, *P* = 1.8 × 10^−54^ for SAHA) (Fig. 2c, d). A weaker, but still statistically significant association was observed between closing DHS sites and decreased nearby gene expression (Mann–Whitney test, *P* = 4.7 × 10^−13^ for NaBut, *P* = 2.2 × 10^−10^ for SAHA). These effects were stronger when considering only those genes that were associated with two or more direction-matched DHS site changes, indicating that these sites have an additive effect on expression (Fig. 2c, d). Together, these results show DHS site opening often marks increases in local transcription levels and support that opened DHS sites are enhancers. Similarly, closing DHS sites may represent reduced distal enhancer activity.

To further test whether HDACi-opened DHS sites function as enhancers in K562 cells, we cloned eight ~800-bp regions encompassing HDACi-opened DHS sites in front of a minimal promoter driving luciferase reporter expression. At 24 h post-transfection into K562 cells, we treated cells with NaBut, SAHA, or their respective vehicle controls for 24 h and then measured luciferase activity. Normalizing signal to co-transfected Renilla luciferase, we detected luciferase activity elevated above the minimal promoter construct alone for five of the eight elements in untreated K562 cells (Fig. 2e, f). Importantly, the addition of either NaBut or SAHA resulted in seven of the eight elements significantly increasing luciferase activity. This effect was not observed in a known strong enhancer that does not increase in accessibility following HDACi treatment (Fig. 2e, f). Together, these results suggest many opened DHS sites indeed function as enhancer elements and that these elements become more active upon HDACi treatment.

### PU.1 binds preferentially at opened DHS sites

**Fig. 3:**
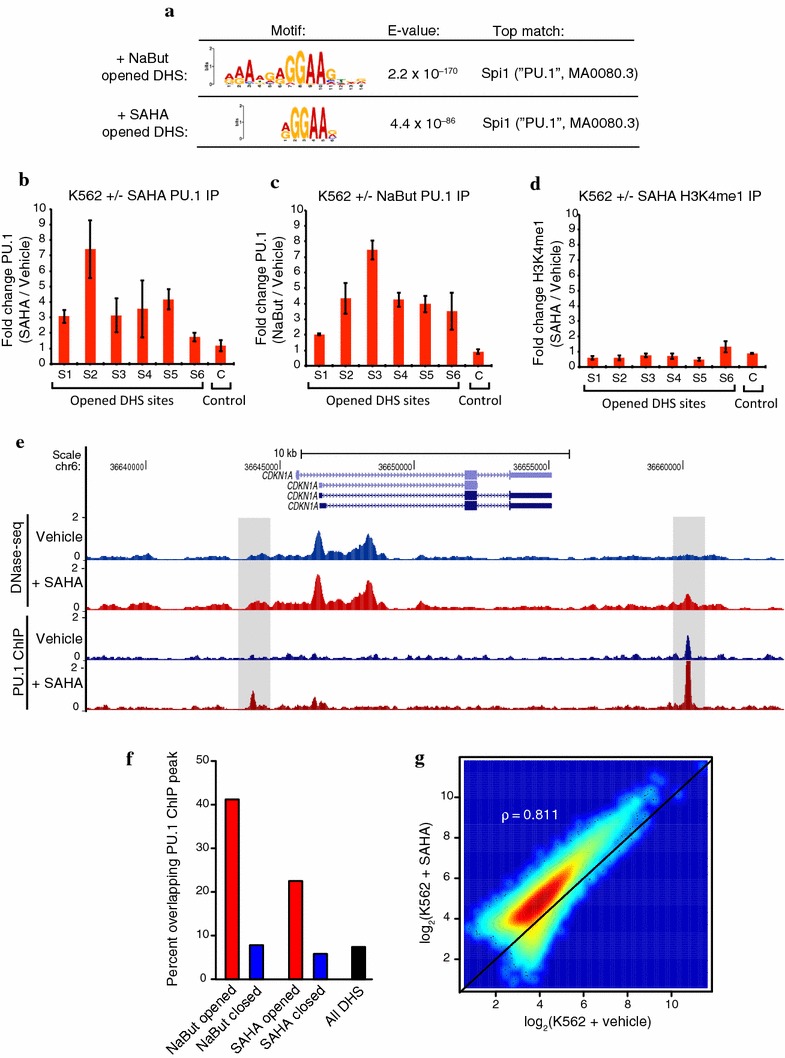
PU.1 increases binding at HDACi-opened DHS sites. **a** PU.1 motif enrichment detected in NaBut and SAHA-opened DHS sites with motif logo, expected (*E*) value, and top JASPAR database motif match found. See Additional file 4: Fig. S2 for all top motif enrichments. ChIP-qPCR was performed for six DHS sites that open in K562 cells following HDACi treatment and a control site that does not change accessibility. The fold-change of PU.1 pull-down enrichment following SAHA (**b**) or NaBut (**c**) 72-h treatment is plotted for each site. *Error bars* are SEM (*n* = 3 replicates). **d** ChIP-qPCR for the same six DHS sites for H3K4me1 immunoprecipitation. The fold-change of H3K4me1 pull-down enrichment following 72-h SAHA treatment is plotted for each site. *Error bars* are SEM (*n* = 3 replicates). **e** Representative example of ChIP-seq data for PU.1 binding in K562 cells before and after 72-h SAHA treatment. The two sites highlighted increase in both DNase and PU.1 binding signal (*n* = 3 replicates). **f** The proportion of differential DHS sites overlapped by PU.1 ChIP-seq peaks. **g** Relationship between total PU.1 ChIP signal found in vehicle controls and SAHA-treated K562 at all PU.1 peaks. ChIP-seq signal is normalized by total number of mapped reads in each condition (Spearman’s correlation = 0.811)

Motif enrichment analysis of DHS sites that open following NaBut or SAHA treatment revealed an ETS family binding motif that closely matched the position weight matrix for SPI1 (PU.1) and was unique to HDACi-opened DHS sites (Fig. 3a; Additional file 4: Fig. S2). Although the large family of ETS transcription factors have highly similar DNA-binding motifs [19], we found PU.1 was the only ETS factor with a significant increase in expression level following both NaBut and SAHA treatments in our RNA-seq data (Additional file 5: Table S3). PU.1 has also been described as a pioneer factor in macrophage and B lymphocyte differentiation with the ability to establish cell lineage-specific enhancer elements by binding and recruiting cofactors and chromatin remodeling proteins [16, 17]. We therefore hypothesized PU.1 may play a similar role in establishing functional enhancer elements in K562 cells at newly opened DHS sites following HDACi treatment.

We first tested PU.1 binding by chromatin immunoprecipitation qPCR (ChIP-qPCR) at six DHS sites that both open with HDACi treatment and contain at least one canonical 5′-GGAA-3′ PU.1-binding motif. Enrichment of PU.1 occupancy was detected for each of the six tested sites (Additional file 6: Fig. S3A, B), and substantial increases in PU.1 occupancy were observed following either SAHA (Fig. 3b) or NaBut (Fig. 3c) 72-h treatments. PU.1 binding and establishment of functional enhancers has been previously reported to coincide with increased local deposition of the histone enhancer mark H3K4me1 [16]. We detected H3K4me1 enrichment at each of the six sites prior to HDACi treatment (Additional file 6: Fig. S3C) that modestly decreased at five of the six sites following treatment (Fig. 3d), possibly reflecting nucleosome repositioning.

To characterize genome-wide PU.1-binding changes with HDACi treatment, ChIP-seq was performed on vehicle and SAHA-treated K562 cells in triplicate, which identified 31977 PU.1-binding sites in total. We observed hundreds of sites with increased PU.1 binding following HDACi treatment similar to those flanking the CDKN1A gene (p21) (Fig. 3e), a well-studied tumor suppressor that mediates p53-dependent G1 growth arrest and becomes up-regulated following HDACi treatment [20]. As predicted, PU.1 binding was highly enriched in opened DHS sites relative to all DHS sites in K562 cells or closed DHS sites (Fig. 3f). Interestingly, PU.1 ChIP-seq signal increased globally at the vast majority of peaks following SAHA treatment (Fig. 3g) while maintaining a similar distribution of total binding sites. This result was detected in each of the three replicates processed as pairs of HDACi-treated and vehicle control cells. The global increase in PU.1 binding also matches our ChIP-qPCR results (Fig. 3b, c).

### PU.1 overexpression modestly increases accessibility

**Fig. 4:**
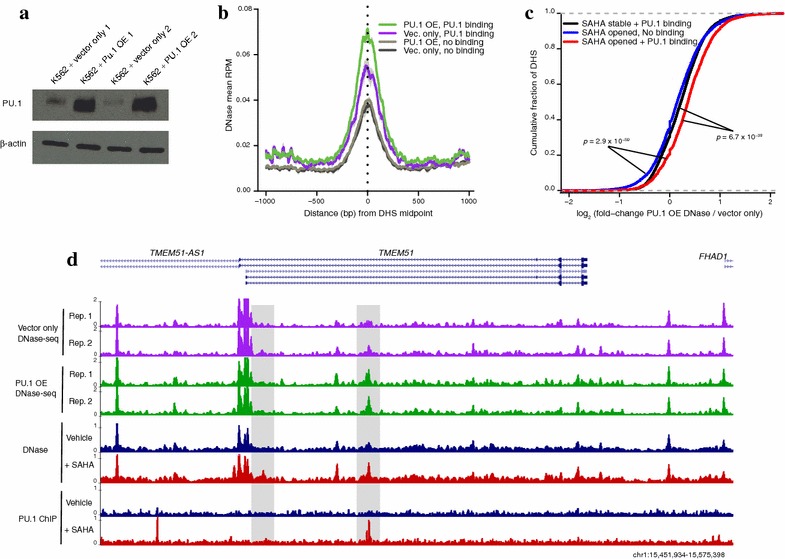
PU.1 overexpression drives modest chromatin accessibility changes. **a** Western blot showing PU.1 overexpression (OE) produces a protein of the same size as endogenous PU.1. β-actin used as loading control (*n* = 2 replicates). **b** PU.1 overexpressing and vector control K562 mean DNase-seq signal found ±1 kb from DHS site center for all SAHA-opened DHS sites split into those that contain a PU.1 binding site (ChIP-seq peak) and those that do not. *Light gray* shading = SEM (*n* = 2 replicates). **c** PU.1-bound, SAHA-opened DHS sites exhibit greater mean fold-change of DNase-seq signal upon PU.1 overexpression than PU.1-bound sites that do not open with SAHA or SAHA-opened DHS sites without PU.1 binding. The cumulative fraction of DHS sites with each level of accessibility change is depicted. Mann–Whitney tests used to assess significance of the shift in distributions. **d** Two example DHS sites (*shaded* in *gray boxes*) found in an intron of *TMEM51*. The first (*left*) represents a SAHA-opened DHS site that does not have a PU.1 ChIP peak and exhibits no increase in accessibility following PU.1 overexpression. The second (*right*) represents a SAHA-opened DHS site that does overlap PU.1 binding and exhibits a modest increase in accessibility following PU.1 overexpression

In addition to our ChIP-seq showing global increases in PU.1 occupancy following SAHA treatment, we also detected that PU.1 expression levels increase during HDACi exposure in our RNA-seq data (Additional file 7: Fig. S4A). PU.1 transcription is known to be tightly regulated during normal hematopoietic differentiation with different expression levels facilitating key transition points in cell lineage [21, 22]. To test whether HDACi-induced up-regulation of PU.1 was responsible for the chromatin accessibility changes, we transfected K562 cells with either a PU.1 cDNA under control of a viral promoter or an empty vector control. We selected for transformed cells by G418 resistance and performed DNase-seq. On the day of cell harvest, we confirmed PU.1 overexpression by qPCR (Additional file 7: Fig. S4B) and western blot (Fig. 4a).

To characterize the impact of overexpression of PU.1 on chromatin accessibility, we analyzed the original set of 7962 SAHA-opened DHS sites and divided them by those bound by PU.1 (*n* = 2137) versus the remaining that did not bind PU.1 (*n* = 5825). For the 2137 sites bound by PU.1, we observed a reproducible increase in mean accessibility in cells that overexpress PU.1 (Fig. 4b). This increase in accessibility was not detected at the 5825 SAHA-opened DHS sites that do not bind PU.1 (Fig. 4b). Furthermore, the distribution of fold-changes in accessibility found at SAHA-opened DHS sites with PU.1 binding was significantly greater than that of the SAHA-opened DHS sites without PU.1 binding (Mann–Whitney test, *P* < 2.9 × 10^−59^) or PU.1-bound DHS sites that did not open further with SAHA treatment (*P* < 6.7 × 10^−39^) (Fig. 4c). These patterns are exemplified by two DHS sites found in the same intron of the TMEM51 gene; both sites open with SAHA treatment, but only the site with PU.1 bound displays increased hypersensitivity following PU.1 overexpression (Fig. 4d). The difference between PU.1-bound DHS sites that open and those that do not increase accessibility with SAHA treatment cannot be explained by differing baseline levels of PU.1 binding (Additional file 7: Fig. S4C). Together these analyses suggest a subset of PU.1 bound DHS sites become more accessible in response to elevated PU.1 expression and that this subset overlaps many of the same DHS sites that open up in response to HDACi treatment.

### Depletion of PU.1 fails to block HDACi-induced chromatin accessibility and expression changes

**Fig. 5:**
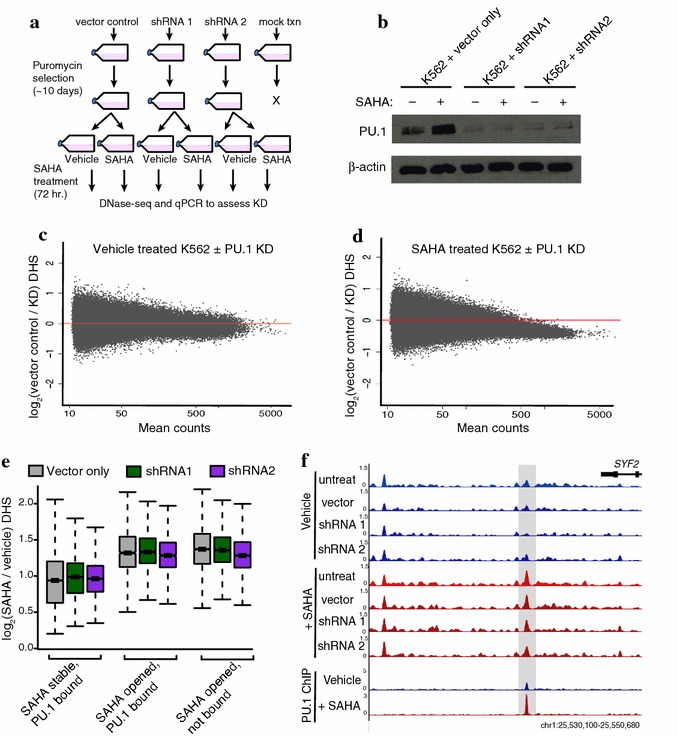
PU.1 depletion fails to block HDACi-induced chromatin accessibility changes. **a** Schematic of PU.1 shRNA knockdown strategy. K562 cells were transfected with a control vector, a vector containing an shRNA that targets *PU.1*, or mock transfected. Following 10 days of puromycin selection, stable knockdown cultures were obtained and immediately subjected to SAHA or vehicle control 72-h treatment followed by DNase-seq and RNA-seq profiling. **b** Western blot showing PU.1 levels before and after SAHA treatment for vector control-, shRNA 1-, or shRNA 2-transfected K562. β-actin was used as loading control (*n* = 2 replicates). **c** MA plot of fold-change in chromatin accessibility (DNase-seq) between vector control and PU.1 knockdown (shRNA 1) K562 over average signal found at each site. There are no significant DHS sites at *P* < 0.10 (*n* = 2 replicates). **d** MA plot of fold-change in chromatin accessibility between vector control and PU.1 knockdown (shRNA 1) K562 following 72-h SAHA treatment over average signal found at each site. There are no significant DHS sites at *P* < 0.10 (*n* = 2 replicates). **e** Boxplots of fold-change in chromatin accessibility between vehicle control and SAHA-treated K562 with vector control or shRNA knockdown of PU.1. Three sets of DHS sites are shown for comparison—PU.1-bound (overlaps ChIP-seq peak) sites that did not open in original SAHA treatment, PU.1-bound sites that open with original SAHA treatment, and unbound sites that open with original SAHA treatment. **f** Representative DHS site located downstream of the *SYF2* gene that opens with original 72-h SAHA treatment (DNase-seq) and contains a PU.1 ChIP-seq peak. The DHS site still opens in PU.1 shRNA knockdown cells

To determine whether PU.1 is required for the chromatin accessibility increases observed with HDACi treatments, we reduced PU.1 levels using shRNA knockdown. Two shRNA constructs that target PU.1 mRNA and a non-hairpin control vector were transfected into K562, and stable transformants were selected with puromycin (Fig. 5a). After selection, cells were treated with SAHA or vehicle control for 72 h and processed for DNase-seq. Reduced PU.1 expression was confirmed on the day of cell harvest by qPCR (Additional file 8: Fig. S5A) and western blot (Fig. 5b).

Surprisingly, depletion of PU.1 displayed no significant differences in chromatin accessibility from control untreated K562 at an adjusted *P* value cutoff of 0.10 (Fig. 5c; Additional file 8: Fig. S5B). These results indicate that normal PU.1 levels are not required for maintaining chromatin accessibility patterns in K562 cells, though we cannot rule out that residual low PU.1 protein levels are sufficient for the chromatin maintenance. Even more surprisingly, we found no significant chromatin accessibility differences in PU.1-depleted versus control cells when treated with SAHA for 72 h (Fig. 5d; Additional file 8: Fig. S5C). We also detected no substantial differences in DNase signal in the subset of PU.1-bound DHS sites that open upon SAHA treatment (Fig. 5e, f; Additional file 8: Fig. S5D, E). This indicates that reduced levels of PU.1 do not prevent the altered chromatin accessibility patterns observed after SAHA treatment.

To determine whether PU.1 is required for the gene expression changes that occur with HDACi treatment, we performed RNA-seq on control and PU.1-depleted cells treated with or without SAHA. We identified just 45 genes in untreated cells and 115 genes in SAHA-treated cells significantly differential (FDR < 0.05) with PU.1 knockdown (Additional file 9: Table S4). Of these, 75.6 % (34/45) and 67.8 % (78/115) were also genes differential as a result of SAHA treatment in our original RNA-seq analysis. This suggests that PU.1 knockdown impacts a fraction of SAHA transcriptional changes in K562. However, the distribution of changes in expression for genes differential upon SAHA treatment in vector control-transfected cells (999 genes at FDR < 0.05) was remarkably similar to cells with PU.1 depleted (Additional file 10: Fig. S6A). Furthermore, a heatmap of these significantly differential genes shows a clear separation between all SAHA-treated samples and all vehicle controls (Additional file 10: Fig. S6B). These results indicate that PU.1 is not required for the vast majority of HDACi-induced expression changes and further suggest other cofactors may be responsible for activation of opened enhancer elements.

### Histone marks and transcription factor binding are predictive of HDACi response at PU.1-bound DHS sites

**Fig. 6:**
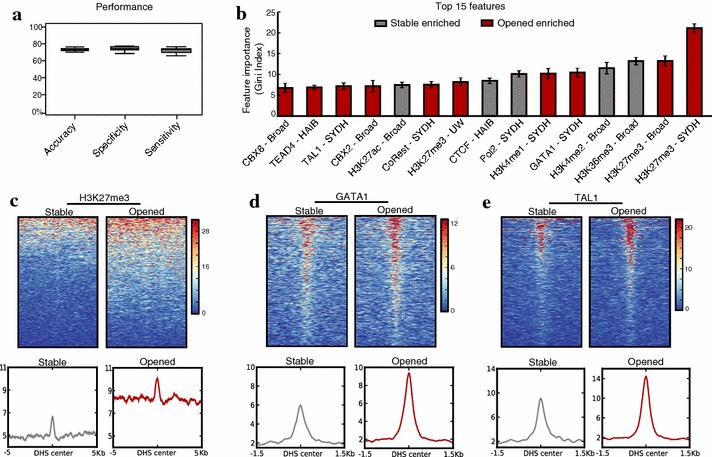
Chromatin features distinguish HDACi-opened from stable DHS sites bound by PU.1. **a** Performance of random forest classifier in distinguishing PU.1-bound DHS sites that remain stable in accessibility from those that significantly open. Plot shows accuracy, specificity, and sensitivity for 10 separate runs with 75 % of input data used for training and the remaining 25 % used for testing. **b** Ranking of top 15 features by Gini index. The same factor may appear more than once if data are available from multiple ENCODE centers. *Broad* Broad Institute, *HAIB* Hudson Alpha Institute for Biotechnology, *UW* University of Washington, *SYDH* Stanford, Yale, Davis, Harvard. Full results can be found in Additional file 12: Table S5. **c**–**e** Top positive predictors of PU.1-bound DHS site opening are shown as heatmaps of ChIP-seq signal present at each DHS site (DHS center ±5 kb for H3K27me3 and ±1.5 kb for TFs). Mean ChIP-seq signal in the same intervals plotted below

To investigate what additional transcription factors or histone modifications might be required for HDACi-induced chromatin opening and enhancer activation at PU.1-bound sites, we leveraged 112 transcription factor, 15 histone modification, and 18 chromatin-modifying factor ChIP-seq datasets available in untreated K562 cells from the Encyclopedia of DNA Elements (ENCODE) project [23]. We used a random forest classifier to identify factors or histone marks most informative for discriminating between PU.1-bound DHS sites that open with SAHA treatment and those that remain stable in accessibility. On ten separate classification runs with 75 % of input data randomly chosen to train and the remaining 25 % used for testing, we found the performance was consistently better than chance (mean accuracy = 72.5 %) and the ranking of the top features was stable (Fig. 6a, b). The analysis identified H3K27me3 as the top positive predictor, while GATA1 and TAL1 appeared as top activating transcription factors predictive of site opening (Fig. 6b).

Heatmaps of H3K27me3, GATA1, and TAL1 ChIP signal over the DHS sites used for classification show these three features are indeed enriched in PU.1-bound DHS sites that open with HDACi treatment (Fig. 6c–e). RNA-seq shows TAL1 and GATA1 significantly increase in expression following SAHA treatment (Additional file 3: Table S2) and the enriched binding of these hematopoietic factors implicates them as regulators of the HDACi-induced enhancers. Interestingly, the presence of elevated H3K27me3 suggests PU.1-bound sites that open with HDACi may be poised in chromatin status for this response [24], while sites stable in accessibility may represent mainly already active enhancers, as evidenced by greater H3K36me3, H3K4me2, RNA polymerase II (Pol2), and H3K27ac signal (Fig. 6b) [25–27]. While not informative for distinguishing between opened and unchanged DHS sites, H3K4me1 signal also appears enriched in all PU.1-bound DHS sites ahead of HDACi treatment (Additional file 11: Fig. S7), consistent with our ChIP-qPCR data (Additional file 6: Fig. S3C).

## Discussion

HDAC inhibitors have the ability to halt proliferation, induce cell death, or resensitize many cancers, but the pleiotropic effects of global hyperacetylation may limit their therapeutic potential. Isolating the processes underlying anticancer efficacy of HDACi from those that are harmful to normal cells is paramount to developing safer, more targeted therapies. We investigated the process of HDACi-induced cancer cell differentiation using K562 cells that slow in growth and display markers of erythroid lineage commitment with treatment [14, 28]. While histone acetylation levels increase genome wide following HDACi treatment [6, 7], our data showed only about 10 % of DHS sites change in K562 cells in response to treatment, with both opening and closing events. This implies that global hyperacetylation of histone tails is not sufficient to appreciably open local chromatin structure. These results are compatible with our RNA-seq data and previous expression studies identifying limited sets of differentially expressed genes following HDACi exposure [11, 12]. We postulate that the chromatin accessibility changes we observed were largely due to programmed enhancer element activation or repression as K562 cells undergo differentiation. The specificity of enhancer activity changes likely results from an exchange of specific transcription factors that either activate and increase accessibility or deactivate and decrease accessibility. While it appears HDACi treatment is capable of gene repression, it may be the case that the DHS sites that close following HDACi treatment represent secondary effects of increased repressive transcription factor expression.

The reprogramming of K562 by HDACi-induced differentiation may be very similar to the mechanism by which all-*trans* retinoic acid is used to treat acute promyelocytic leukemia (APL). In APL, all-*trans* retinoic acid induces differentiation of leukemic promyelocytes into mature granulocytes [13]. Chromosomal rearrangements in APL most commonly fuse the retinoic acid receptor-alpha (*RARA*) gene to the promyelocytic leukemia (*PML*) gene, generating a hybrid protein that binds to genes involved in normal granulocyte differentiation and recruits the nuclear corepressor (NCoR) complex [29]. NCoR represses gene expression by recruiting HDACs, and all-*trans* retinoic acid alleviates this repression by effectively displacing NCoR. Indeed, this connection between HDAC-mediated gene repression in APL and the mechanism of HDACi treatments has been suggested before [30]. Our evidence of specific chromatin and gene expression changes driving K562 differentiation in the presence of HDACi support the idea that similar complexes may repress tumor suppressor genes, and this repression is alleviated after treatment.

Our motif enrichment analysis and expression data implicated the pioneer factor PU.1 in the HDACi-induced opening of DHS sites. ChIP-seq for PU.1 before and after treatment demonstrated that this transcription factor binds many of the opened DHS sites and increases binding following HDACi treatment. PU.1 is known to be critical for enhancer activation and chromatin changes in B cell and macrophage differentiation [16, 17]. However, we found evidence that PU.1 is not a major driver of HDACi enhancer activation in K562 cells. Forced overexpression of PU.1 only produced modest chromatin accessibility increases at a subset of bound loci, and reduction of PU.1 levels by shRNA knockdown did not prevent the chromatin accessibility increases or gene expression changes with HDACi treatment. However, we cannot rule out PU.1 may be still acting as a pioneer factor earlier during the development of the K562 epigenome and more important for poising future enhancer sites. It is also possible that low residual PU.1 levels are enough to initiate the process of chromatin remodeling and that increased binding at opened DHS sites is not important for this process. Creating a PU.1 null cell line may help address this possibility. Lastly, it may be the case that other factors, including ETS family proteins, are functionally redundant in the chromatin remodeling process and capable of assuming PU.1’s enhancer activation role in K562.

Using a random forest classifier, we identified the histone mark H3K27me3 as the strongest positive predictor for PU.1-bound DHS sites to respond to HDACi treatment with chromatin accessibility increases. Additionally, we found that the chromobox proteins CBX2 and CBX8, which are components of Polycomb repressor complexes that maintain H3K27me3 marks [31], are enriched at the responsive DHS sites (Fig. 6b; Additional file 11: Fig. S7B, C). The dual presence of H3K27me3 and H3K4me3 has been described as “bivalent domains” with competing activating and repressive modifications that together mark poised promoter regions during development [24]. As cells differentiate, they can potentially activate or repress marked genes by changing histone modifications and chromatin accessibility status. Similarly, H3K4me1 in combination with H3K27me3 marks poised distal enhancer elements [32]. The presence of the H3K4me1 and H3K27me3 marks, together with the low levels of PU.1 binding prior to HDACi treatment, suggests the HDACi-opened DHS sites we observed might be initially poised for enhancer activity and a chromatin remodeling response in K562 cells. In this model, once de-repressed by removal of HDAC function, these enhancers become activated and drive gene expression patterns that coordinate the erythroid differentiation process. The PU.1-bound DHS sites that do not significantly increase accessibility with HDACi treatment likely include more sites that are already active enhancers, as evidenced by enriched H3K36me3, Pol2, H3K4me2, and H3K27ac signal (Fig. 6b) [25–27].

We also found a moderate enrichment of GATA1 and TAL1 binding at opening PU.1-bound DHS sites. The GATA family motif was enriched in our sets of HDACi-opened and HDACi-closed DHS sites in general (Additional file 4: Fig. S2). PU.1 and GATA1 are capable of inhibiting each other’s function by protein–protein interactions that block DNA binding [33, 34], but it is unclear how that mechanism may be involved in this process. TAL1 has been shown to be required for chromatin looping between the β-globin locus control region and the γ-globin gene to enhance its expression in K562 cells, demonstrating a role it plays in this system to establish functional enhancers [35]. The extent to which PU.1, GATA1, and TAL1 cooperatively interact to control enhancer activation following HDACi treatment is currently unknown and will be the target of future studies. As the role of lineage-committing transcription factors and chromatin changes in induced cancer cell differentiation are better defined, strategies can be further refined to inform more targeted cancer treatments.

## Conclusions

We found that despite widespread hyperacetylation, HDACi cause site-specific chromatin remodeling in the genome of K562 cells with roughly equal numbers of DHS sites gaining or losing accessibility. Opening DHS sites often reflect gain of enhancer activity at sites marked by PU.1 binding that increases with HDACi treatment. PU.1-bound DHS sites with the largest accessibility gains appear to be epigenetically poised by a combination of repressive H3K27me3 and activating H3K4me1 histone marks. Together, these results help explain the ability of HDACi to drive differentiation of K562 cells at sublethal concentrations by activating and deactivating particular enhancer elements to regulate gene expression in a pre-programmed manner. We postulate a similar directed differentiation process underlies HDACi efficacy against various leukemias and contributes to the ability of HDACi to sensitive cancer cells to other drugs in combination therapies.

## Methods

### K562 cell culture and HDACi treatments

K562 cells were obtained from ATCC (CCL-243) and maintained in 1× RPMI1640 media supplemented with 10 % FBS and 1× antibiotic–antimycotic (Life Technologies). Initial titrations of the HDACi NaBut and SAHA (Sigma-Aldrich) were performed to determine the maximum concentration with which <10 % of cells are killed at 72 h by Trypan blue staining. Addition of NaBut dissolved in 1× PBS to 0.5 mM or SAHA dissolved in DMSO to 1 μM final concentration versus an equal volume of vehicle was used for all 72-h exposures presented with the exception of 2 mM NaBut presented in Additional file 1: Fig. S1. Additionally, 1 mM NaBut was used for the shorter 24-h treatments with luciferase reporter constructs.

### DNase-seq

15–50 million K562 nuclei were spun down, washed twice with 1× PBS, divided, and digested with a range of recombinant DNase I enzyme (Roche) concentrations (between 0.12 and 12 U) for 16 min at 37 °C in 120 μL 1× DNase buffer. DNase-seq libraries were constructed as previously described [36]. Briefly, each set of digestions was checked by pulsed-field gel electrophoresis, and material was pooled in equimolar amounts following blunt-ending reactions from three different DNase concentrations to match extent of digestion (0.36, 1.2, and 3.6 U used in all experiments). Following ligation to adapters, MmeI digestion, streptavidin bead-based enrichment, and 14 cycles of linker-mediated PCR, each library was sequenced for 50 cycles on the Illumina HiSeq2000 platform, with the exception of the first replicate of NaBut treatments, which were sequenced for 36 cycles on a GAIIx machine. Three replicates (beginning with a new K562 frozen stock) were processed per condition with HDACi-treated and corresponding vehicle control samples co-processed throughout each replicate.

DNase-seq reads were trimmed to the first 20 bp (fixed insert length generated by MmeI digest) and aligned to the UCSC hg19 reference genome using Bowtie [37] allowing up to 1 mismatch and requiring unique alignments. Potential PCR artifacts were filtered from alignment by removing reads where greater than 70 % map to a single bp within a 31-bp window [38]. Reads mapping to ENCODE blacklisted regions [23], mitochondria, and chrY were removed from alignment. MACS2 [39] was used to call peaks (FDR < 0.05) and generate genome coverage files by artificially extending DNase reads to 200 bp in length and centering on 5′ read ends. bigWig files for browser images were normalized to millions of reads aligned per sample.

For differential signal tests, raw read counts were tabulated for the union of all peak calls across replicates as input for DESeq2 [18]. Principle components analysis and clustering by Euclidean distance were executed using DESeq2 following regularized log transformation of normalized read counts of DNase signal found in all peaks. DHS sites were associated with the single nearest hg19 reference gene for expression level comparisons. DHS sites mapping within 2 kb of an annotated TSS were considered promoter-located, and all other DHS sites were considered distal. Enriched motifs in differential DHS sites were identified using MEME-ChIP [40] with alignments to JASPAR and UniPROBE databases.

### Chromatin immunoprecipitation

ChIP was performed essentially as previously described [41]. Briefly, 20 million K562 cells per ChIP were cross-linked with 1 % formaldehyde for 10 min, quenched with 0.125 M glycine, and lysed. DNA was sonicated by 45 min with 30-s on/off cycles on a Bioruptor (Diagenode) in RIPA buffer. An aliquot of sonicated material was reserved for agarose gel sizing and for an input chromatin control sample with each treatment. After resuspending chromatin in 1 % Triton X-100, 2 mM EDTA, 20 mM TrisCl, 150 mM NaCl buffer, and pre-clearing material with Protein A Dynabeads (Life Technologies) for 2 h at 4 °C, 6 μg of the following antibodies was incubated with the sonicated chromatin overnight at 4 °C: rabbit anti-PU.1 Santa Cruz Biotech sc-22,805 and rabbit anti-H3K4me1 Abcam ab8895. Protein A beads were bound to antibody for 3 h at 4 °C and then washed five times with LiCl wash buffer (500 mM LiCl, 100 mM TrisCl, 1 % NP40, 1 % sodium deoxycholate) and once with 1× TE. Cross-linking was reversed with overnight 65 °C incubation in 1 % SDS and 0.1 M sodium bicarbonate buffer, and DNA was eluted with a PCR cleanup kit (Qiagen). Following TruSeq adapter ligations, DNA fragments were separated from free adapters by AmpureXL bead size selection steps, and PCR amplification was performed for 18 cycles. ChIP-seq libraries were sequenced on the Illumina HiSeq2000 machine with 50-bp SE reads. Three replicates (beginning with a new K562 frozen stock) were processed per condition with HDACi-treated and corresponding vehicle control samples co-processed throughout each replicate to mitigate batch effects.

ChIP-seq reads were aligned to the hg19 reference genome using Bowtie [37] allowing up to 1 mismatch and requiring unique alignments. Putative PCR artifacts and reads mapping to blacklisted regions, mitochondrial, or chrY sequences were removed exactly as with DNase-seq alignments. Peak calls were generated by MACS2 [39] with default settings at FDR < 0.05 over K562 input background samples with matching vehicle or HDACi treatment where appropriate. Genome browser visualizations were normalized by total mapped reads in each sample. Genomic regions tested for enrichment by qPCR were amplified with the following primers: S1, F: ACTTCCCCTTTCCCTTGCT, R: GGGCTGGGAGGACTACTGTG; S2, F: GCCTTGGGCAGATGTACAAA, R: TTCCACTTCCTCTTTCTGTGC; S3, F: ACCAGAGGTCCCTGGAGTG, R: CTGGGAGGACAGCTGCTAAG; S4, F: TCAAGTCGTGGTTTGGATGA, R: GGGGTACCTCTCACCACTCA; S5, F: GATCTTGGGGGTGGCTTG, R: AAGTGAGAAGGGGCTGTTGT; S6, F: GACTGGGGGAAGGACCTCT, R: CCAGCACGAAGCTGACTGAT; C1, F: TCTCTGGGGAGATGGATTACA, R: CGTGAATCCTTTATTCTTGGAA; C2, F: CCAATAACAGAAGCATTAAAATTCA, R: TTCAAGCACAGGCATACAGG.

### RNA-seq

RNA was isolated from 5 to 10 million K562 cells with an RNeasy Mini kit (Qiagen) with on-column DNase digestion. Two microgram of total RNA was used for TruSeq library preparation with polyA selection. Libraries for initial NaBut and SAHA versus vehicle control experiments were subjected to 50-bp paired-end Illumina HiSeq2000 sequencing to an average depth of 40.2 million read pairs. Libraries for SAHA versus vehicle control with PU.1 depleted by shRNA were sequenced as 50-bp SE on Illumina HiSeq with average depth of 36.4 million reads. Following quality-score-based trimming, reads were aligned to hg19 UCSC genes with Tophat v2.0.12 [42] allowing up to 2 mismatches. FPKM estimates for individual samples were obtained with Cufflinks v2.2.1, and differential genes across conditions were identified with Cuffdiff v2.2.1 [43]. Three (for NaBut treatment) or two (for SAHA treatments) independent replicates (beginning with new K562 frozen stock) were processed with HDACi-treated and corresponding vehicle control samples in parallel throughout.

### Classification of opened versus stable sites

In order to identify transcription factors and epigenetic marks associated with increased accessibility of DHS sites bound by PU.1, we implemented a random forest classifier to distinguish sites that open from those that do not significantly change accessibility following HDACi treatment. We used all ChIP-seq data available through ENCODE (genome assembly hg19) for the untreated K562 cell line (“treatment = None”) with the exception of experiments labeled with “revoked” status. A total of 112 transcription factor, 15 histone modification, and 18 chromatin-modifying factor ChIP-seq alignments were downloaded and processed with MACS2 (v2.10) [39] to generate fragment pileup scores (bedGraph format) over input control. Before classification, we removed all DHS sites localizing to known gene promoters or exons (based on UCSC hg19 known genes). Next, to control for the DNase-seq and PU.1-binding signal in the sets of opened versus stable sites used for classification, for each opened DHS site we randomly selected a stable DHS site with close to equal baseline DNase-seq and PU.1 ChIP-seq signal in untreated K562 cells. DHS center ±200 bp was used for DNase signal, and DHS center ±150 bp was used for PU.1 ChIP signal matching. This resulted in ~930 opened and stable sites used for classification. For each selected DHS site, transcription factor features were computed as the maximum ChIP-seq pileup signal over 200-bp windows with 100-bp overlap in the DHS site (defined as DHS site center ±300 bp). For chromatin-modifying factors, we used a larger region of DHS center ±700 bp. For histone modifications, we summed the total ChIP-seq signal in the DHS center ±700 bp. The random forest classifier was run through R package “randomForest” with “mtry” set to 10 and “ntree” set to 500. 75 % of the input data were used to train the model and 25 % reserved for testing. The random splitting of training and testing samples followed by classification was repeated 10 times to assess the stability of the top features and classification accuracy. The importance score of each feature was computed as the Gini index value and the mean decrease in accuracy when its class labels are randomly permuted. Heatmap and summary plots were generated using deepTools software [44].

### Luciferase assays

~800 bp of each DHS site was cloned upstream of the minimal promoter in a *pGL4.23* luciferase reporter vector (Promega) using amplicons generated with the following primers (hg19): chr1:228639823–228640570, F: GATGACTGGGGGAAGTCTCA, R: GACAGCAGGTGTCTGGTGAA; chr11: 76847652–76848380, F: AGTTAACCATGGCTGGCACT, R: GCAACGAAAAAGACGGTGAT; chr19: 8641896–8642579, F: TTGTCCCTGCAGACTCTGTG, R: CTGAATCGCCCTGAAAAGAG; chr5: 95168365–95169111, F: CCATTTCTGCCTCCCTCATA, R: GCGCTTTCCGACTACTCATC; chr6: 41690943–41691723, F: CCTTCTTCCCCATTCTTTCC, R: AGGAAGATGGTGAGCTGTGG; chr19: 6778173–6778776, F: CCTGACCTTAAGTGACCCACA, R: CAGGCAAAATTGGGTTTTGT; chr7: 100839167–100839788, F: AGTGGGTGGAGGTCTCAGTG, R: TGAATGACCCTGGGAGGTAG; chr1: 35384552–35385452, F: TCCTCGTGTCTGCTGTGATG, R: TTTTTGGTCTGGCAGTAGGG; chr9: 131900863–131902480, F: ATCCCTCCTCAGTCCCTTTC, R: AGGGGAATCGTGTGAGTCAG. Inserts were verified by Sanger sequencing. K562 cells were transferred to antibiotic-free media 24-h preceding transfection. 15,000 K562 cells were added per well to 96-well plates and transfected with the *pGL4.23* construct and *pGL4.73Renilla* control plasmid using Lipofectamine LTX with Plus reagent (Thermo Fisher Scientific) following manufacturer’s protocol. 24 h after transfection, 1 mM NaBut, 1 μM SAHA, or an equal volume of 1× PBS or DMSO vehicle was added to each well. 24 h after HDACi addition, cells were simultaneously lysed and exposed to substrate following manufacturer’s instructions for the dual-luciferase reporter system (Promega). Plate emissions were read on a VICTOR2 multilabel counter plate reader (Perkin Elmer). Measurements were performed in technical replicates of six wells, normalized per well by *Renilla* output, and normalized per plate by average empty *pGL4.23* signal. Each construct was tested with at least two separate sets of transfections on different days. The positive control region (chr9: 131900863–131902480) overlaps an alternative promoter of *PPP2R4* that we observed exhibits strong enhancer activity with luciferase reporters in K562 cells, but does not significantly open with HDACi treatment.

### PU.1 shRNA knockdown

We purchased shRNAs targeting human PU.1 mRNA that were cloned in the lentiviral vector *pLKO.1* (Sigma-Aldrich) and obtained a non-hairpin control *pLKO.1* (Addgene plasmid #10879 from David Root). Five shRNAs targeting PU.1 were tested for knockdown efficiency, and the two most effective, TRCN0000426240 and TRCN0000417534, were selected for subsequent use. shRNA vectors were introduced by transfection of 10 million K562 cells with Lipofectamine LTX with Plus reagent (Thermo Fisher Scientific) following manufacturer’s protocol. 24 h after transfection, puromycin (Gibco) was added to media to 2 μg/mL for selection over 10–12 days. Puromycin was reduced to 1 μg/mL during 72-h SAHA treatments. Knockdown efficiency was evaluated by qPCR or western blot on days cells were subjected to DNase-seq.

### PU.1 overexpression

PU.1 cDNA was obtained by reverse transcription from K562 total RNA extracts and amplification with following primers (IDT): F: TGACGGATCC GCCGCCACCATGTTACAGGCGTGCAAAATG, R: TGACGCGGCCGCTCAGTGGGGCGGGTGG that amplify from start to stop codons of PU.1 cDNA with addition of a Kozak sequence, and BamHI and NotI recognition sites. BamHI and NotI double digest (NEB) was used to clone the amplified cDNA into the pTargeT mammalian expression vector (Promega), and the insert was verified with Sanger sequencing. The PU.1 overexpression construct or empty pTarget was introduced to 0.5 million K562 cells by transfection with Lipofectamine LTX with Plus reagent (Thermo Fisher Scientific) following manufacturer’s protocol. 24 h following transfection, media were supplemented with 400 μg/mL G418 disulfate (Sigma-Aldrich) for 12–14 days of selection. Following selection, G418 concentration was reduced to 250 μg/mL and cells were expanded for 3–4 days. Overexpression was verified by qPCR or western blot on days cells were subjected to DNase-seq.

### Quantitative RT-PCR

1–2 μg of total RNA was used for reverse transcription with oligo dT primers and Superscript II (Invitrogen). Quantitative SYBR green PCR was performed on an ABI 7500 real-time PCR machine (Applied Biosystems) using the following primers (IDT): PU.1, F: TGTTACAGGCGTGCAAAATG, R: TCATAGGGCACCAGGTCTTC; β-actin, F: GCCGGGACCTGACTGACTAC, R: TTCTCCTTAATGTCACGCACGAT; CDKN1A, F: GACTCTCAGGGTCGAAAACG, R: GGATTAGGGCTTCCTCTTGG. Each sample was measured in technical triplicate and normalized to β-actin.

### Western blots

5–10 million K562 cells were lysed in RIPA buffer and spun down at 10,000 RPM for 15 min to remove cell debris. Samples were disrupted by brief sonication, a BCA assay (Pierce) was used to measure protein concentrations, and 10 μg of each lysate was loaded per lane for SDS-PAGE. Gels were transferred to nitrocellulose for blotting. Primary antibodies used were rabbit anti-PU.1 Abcam ab76543 and mouse anti-β-actin Santa Cruz Biotech sc-81178. Bands were visualized by supplying substrate for the following HRP-conjugated secondary antibodies: goat anti-rabbit Pierce #31462 and goat anti-mouse Santa Cruz Biotech sc-2005.

## Data access

All sequencing data presented here (with the exception of ENCODE ChIP-seq datasets referenced above) have been deposited as GEO entry GSE74999.

## Additional files

10.1186/s13072-016-0065-5 DESeq2 output for differential accessibility (DNase-seq) following 72-h NaBut or SAHA treatment of K562 cells.
10.1186/s13072-016-0065-5 Chromatin accessibility changes induced by NaBut or SAHA in K562 cells exhibit high concordance. Mean DNase-seq signal found +/− 2 kb from center of each DHS site called significant in DESeq analysis for NaBut opened (a), NaBut closed (b), SAHA opened (c), and SAHA closed (d). DMSO is vehicle control for SAHA treatments and PBS is vehicle control for NaBut treatments. Note the directional concordance between NaBut and SAHA treatments. Gray shading indicates SEM between replicates (*n* = 3 replicates). RPM = Reads per million mapped. (e) MA plot of fold-change in chromatin accessibility (DNase-seq signal) over average signal found at each site following 72-hour treatment of K562 with 2 mM NaBut. Red marks DHS sites with significantly changed chromatin accessibility (FDR < 0.05, n = 2 replicates). (f) Heatmap of Euclidean distance between regularized log-transformed DNase-seq data for vehicle control K562 (DMSO or PBS) and HDACi-treated K562 (0.5 mM NaBut, 2 mM NaBut, or 1 uM SAHA). Note the agreement between replicates and the clustering of HDACi-treated vs. untreated samples.
10.1186/s13072-016-0065-5 Cuffdiff output for differentially expressed (RNA-seq) genes following 72-hour NaBut or SAHA treatment of K562 cells.
10.1186/s13072-016-0065-5 Motif enrichment in differential DHS sites. The top four enriched motifs (MEME-ChIP results) found in opened or closed DHS sites are listed with corresponding position weight matrix, expected (E) value, and top factor match found in the JASPAR or UniPROBE databases.
10.1186/s13072-016-0065-5 RNA-seq expression data for ETS family transcription factors detected in K562 cells.
10.1186/s13072-016-0065-5 PU.1 and H3K4me1 immunoprecipitation. ChIP-qPCR for six DHS sites that open in K562 cells following HDACi treatment and a control site that does not change accessibility. PU.1 enrichment before and after 72-hour (a) SAHA or (b) NaBut treatment and (c) H3K4me1 enrichment before and after SAHA treatment for each site was measured. Enrichment is normalized to that measured in respective input control samples. Error bars are SEM (n = 3 replicates).
10.1186/s13072-016-0065-5 PU.1 overexpression-driven chromatin accessibility changes are not linked to initial PU.1 ChIP peak strength. (a) *PU.1* expression levels measured by RNA-seq FPKM before and after HDACi treatments. Error bars are SEM (n = 2 replicates for SAHA, 3 replicates for NaBut). (b) Fold-change in expression level of *PU.1* measured by qPCR following PU.1 overexpression construct transfection and selection. Expression normalized to β-actin. Error bars are SEM (n = 3 replicates). (c) Distribution of PU.1 ChIP-seq signal found within 1 kb of DHS site center for SAHA-opened DHS sites that overlap PU.1 ChIP peaks and SAHA-unchanged (stable) DHS sites that overlap PU.1 ChIP peaks. Curves with lower mean are PU.1 signal for vehicle treated K562, and curves with greater mean are PU.1 signal for SAHA-treated K562. Signal is average of three replicates.
10.1186/s13072-016-0065-5 PU.1 knockdown fails to block most HDACi-induced chromatin accessibility changes in K562. (a) *PU.1* expression levels after 72-hour SAHA exposure for vector control-, shRNA 1-, and shRNA 2-transfected K562 cells measured by qPCR. Expression normalized to β-actin. Error bars are SEM (n = 3 replicates). (b) MA plot of fold-change in chromatin accessibility (DNase-seq) between vector control and PU.1 knockdown (shRNA 2) K562 over average signal found at each site. There are no significant DHS sites at *P* < 0.10 (n = 2 replicates). (c) MA plot of fold-change in chromatin accessibility between vector control and PU.1 knockdown (shRNA 2) K562 following 72-hour SAHA treatment over average signal found at each site. There are no significant DHS sites at *P* < 0.10 (n = 2 replicates). (d) Mean DNase-seq signal for vector control or PU.1 knockdown K562 in original SAHA-opened DHS sites that overlap a PU.1 ChIP-seq peak. RPM = reads per million mapped. (n = 2 replicates for vector control, n = 4 replicates for shRNA 1 and 2 combined). (e) Mean DNase-seq signal for vector control or PU.1 knockdown K562 in original SAHA-opened DHS sites that do not contain a PU.1 binding site. RPM = reads per million mapped. (n = 2 replicates for vector control, n = 4 replicates for shRNA 1 and 2 combined).
10.1186/s13072-016-0065-5 Cuffdiff output for differentially expressed (RNA-seq) genes following PU.1 shRNA knockdown in K562 cells.
10.1186/s13072-016-0065-5 PU.1 depletion fails to block HDACi-induced gene expression changes in K562. (a) Boxplots of fold-change in RNA-seq expression for SAHA treatment of vector control-, shRNA 1-, or shRNA 2-transfected cells (n = 2 replicates) for genes that either increase or decrease significantly in vector control K562 (FDR < 0.05). (b) Heatmap of individual replicate expression values (FPKM values scaled across each row) for genes significantly differential following SAHA treatment in vector control K562. Note the distinction between all vehicle treated and all SAHA-treated samples and lack of distinction between PU.1 knockdown and vector control samples.
10.1186/s13072-016-0065-5 H3K4me1 enrichment and CBX2/8 binding at PU.1-bound DHS sites. Heatmaps of ChIP-seq signal present at each DHS site (DHS center +/− 5 kb) for the H3K4me1 mark (a), and chromobox proteins CBX2 (b) and CBX8 (c). Mean ChIP-seq signal present in the same regions plotted below. Note that H3K4me1 is similarly enriched in both opened and stable PU.1-bound DHS sites, while CBX2 and CBX8 display stronger signal in a subset of opened DHS sites and are therefore informative features for the random forest analysis presented in Fig. 6.
10.1186/s13072-016-0065-5 Feature importance scores according to a random forest classification of PU.1-bound opened vs. stable DHS sites.
