# Supplementary figures and images for: HDAC inhibitors cause site-specific chromatin remodeling at PU.1-bound enhancers in K562 cells
Source: Epigenetics Chromatin. 2016 Apr 16;9:15. doi: 10.1186/s13072-016-0065-5 (PMC4833939; doi:10.1186/s13072-016-0065-5)

# Supplementary Figure 1

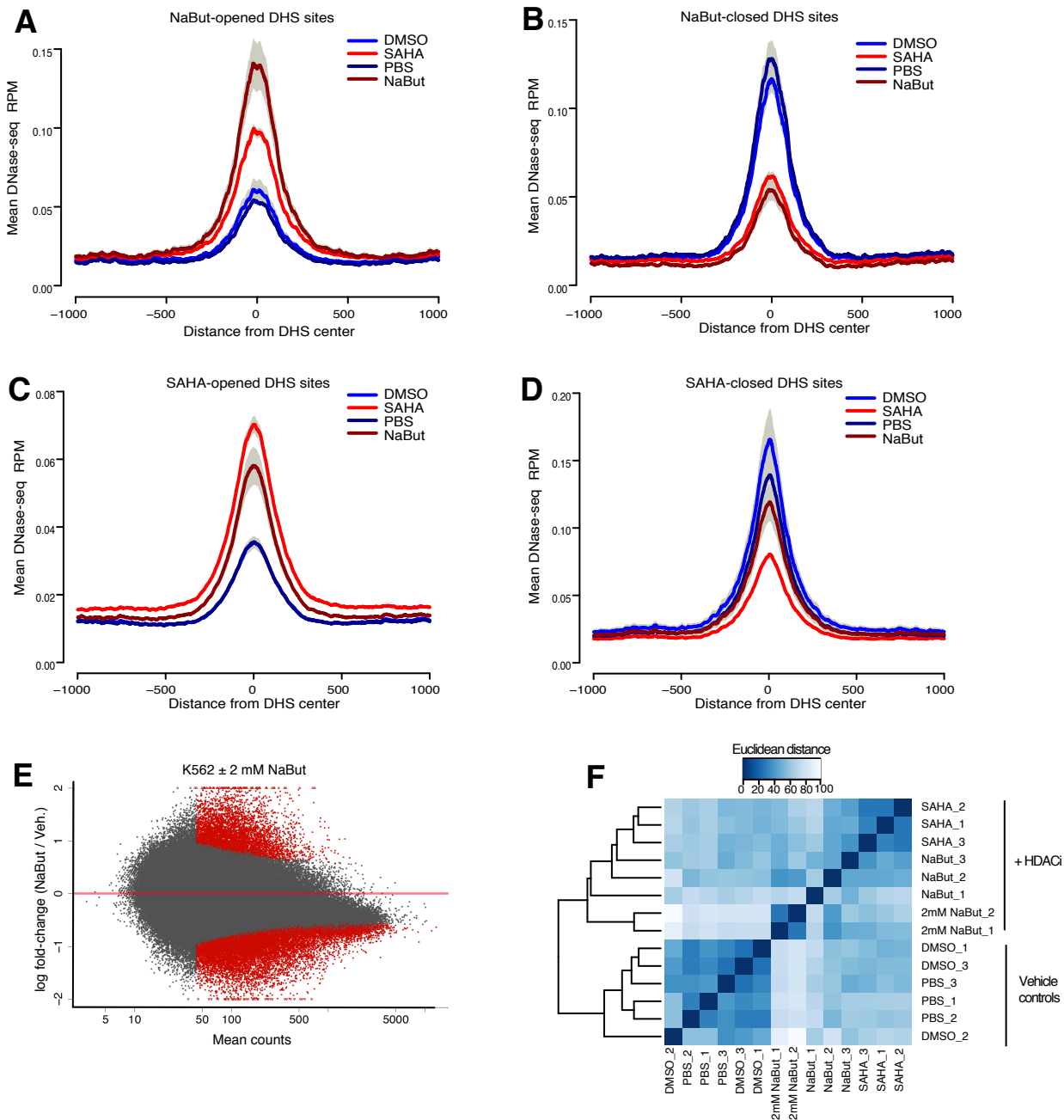

Supplement: Supplementary file 2 — 10.1186/s13072-016-0065-5 Chromatin accessibility changes induced by NaBut or SAHA in K562 cells exhibit high concordance. Mean DNase-seq signal found +/− 2 kb from center of each DHS site called significant in DESeq analysis for NaBut opened (a), NaBut closed (b), SAHA opened (c), and SAHA closed (d). DMSO is vehicle control for SAHA treatments and PBS is vehicle control for NaBut treatments. Note the directional concordance between NaBut and SAHA treatments. Gray shading indicates SEM between replicates (n = 3 replicates). RPM = Reads per million mapped. (e) MA plot of fold-change in chromatin accessibility (DNase-seq signal) over average signal found at each site following 72-hour treatment of K562 with 2 mM NaBut. Red marks DHS sites with significantly changed chromatin accessibility (FDR < 0.05, n = 2 replicates). (f) Heatmap of Euclidean distance between regularized log-transformed DNase-seq data for vehicle control K562 (DMSO or PBS) and HDACi-treated K562 (0.5 mM NaBut, 2 mM NaBut, or 1 uM SAHA). Note the agreement between replicates and the clustering of HDACi-treated vs. untreated samples. [file 13072_2016_65_MOESM2_ESM.pdf]

# Supplementary Figure 3

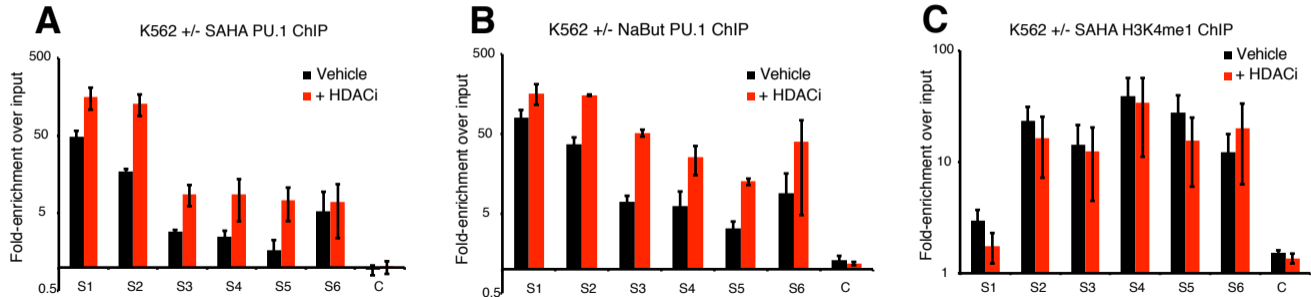

Supplement: Supplementary file 6 — 10.1186/s13072-016-0065-5 PU.1 and H3K4me1 immunoprecipitation. ChIP-qPCR for six DHS sites that open in K562 cells following HDACi treatment and a control site that does not change accessibility. PU.1 enrichment before and after 72-hour (a) SAHA or (b) NaBut treatment and (c) H3K4me1 enrichment before and after SAHA treatment for each site was measured. Enrichment is normalized to that measured in respective input control samples. Error bars are SEM (n = 3 replicates). [file 13072_2016_65_MOESM6_ESM.pdf]

# Supplementary Figure 4

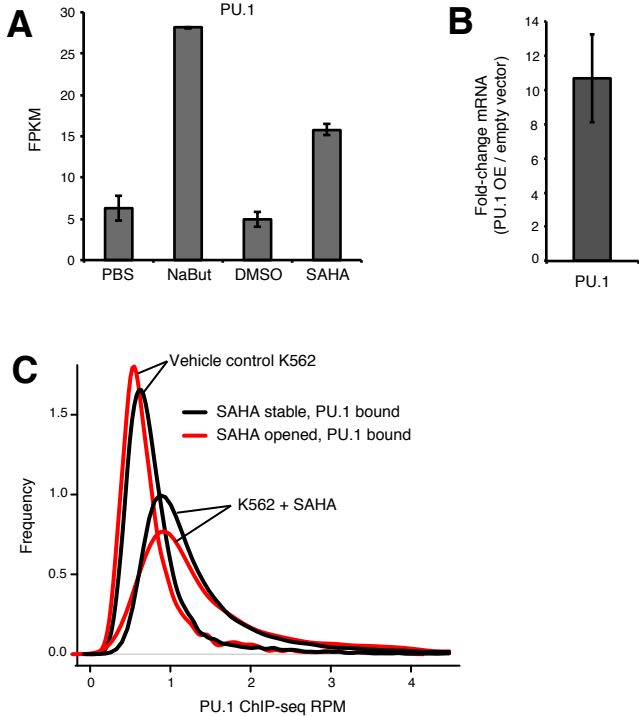

Supplement: Supplementary file 7 — 10.1186/s13072-016-0065-5 PU.1 overexpression-driven chromatin accessibility changes are not linked to initial PU.1 ChIP peak strength. (a) PU.1 expression levels measured by RNA-seq FPKM before and after HDACi treatments. Error bars are SEM (n = 2 replicates for SAHA, 3 replicates for NaBut). (b) Fold-change in expression level of PU.1 measured by qPCR following PU.1 overexpression construct transfection and selection. Expression normalized to β-actin. Error bars are SEM (n = 3 replicates). (c) Distribution of PU.1 ChIP-seq signal found within 1 kb of DHS site center for SAHA-opened DHS sites that overlap PU.1 ChIP peaks and SAHA-unchanged (stable) DHS sites that overlap PU.1 ChIP peaks. Curves with lower mean are PU.1 signal for vehicle treated K562, and curves with greater mean are PU.1 signal for SAHA-treated K562. Signal is average of three replicates. [file 13072_2016_65_MOESM7_ESM.pdf]

# Supplementary Figure 5

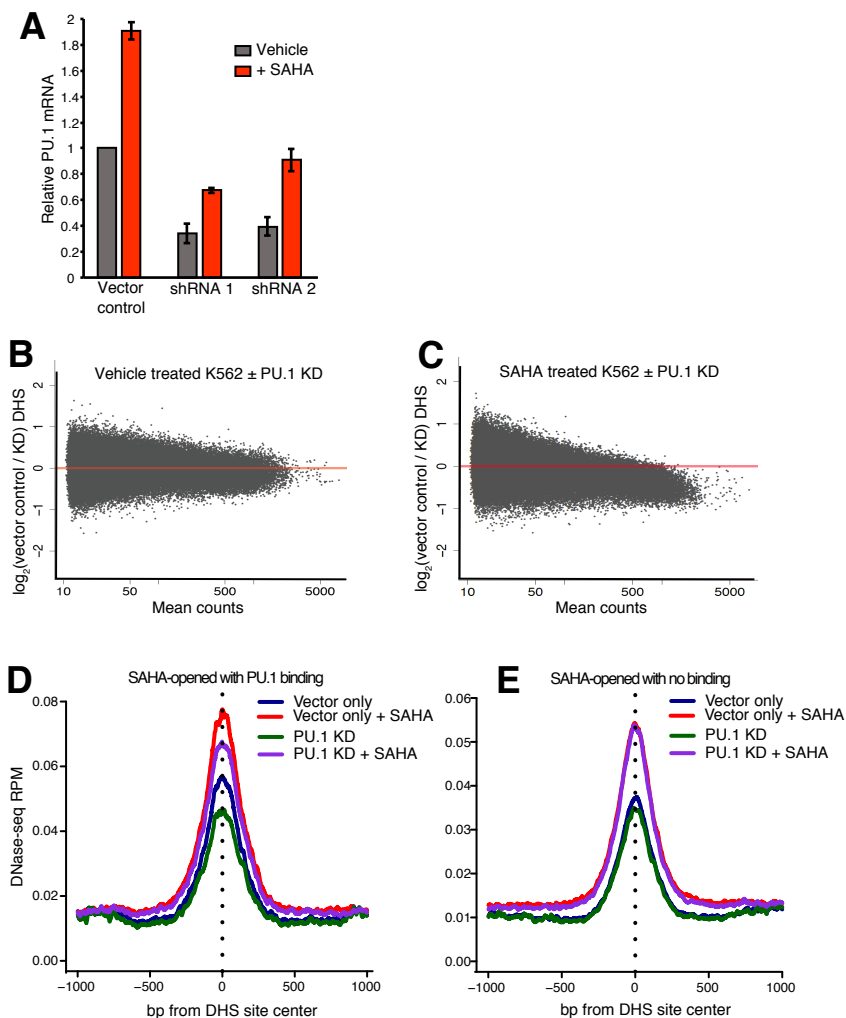

Supplement: Supplementary file 8 — 10.1186/s13072-016-0065-5 PU.1 knockdown fails to block most HDACi-induced chromatin accessibility changes in K562. (a) PU.1 expression levels after 72-hour SAHA exposure for vector control-, shRNA 1-, and shRNA 2-transfected K562 cells measured by qPCR. Expression normalized to β-actin. Error bars are SEM (n = 3 replicates). (b) MA plot of fold-change in chromatin accessibility (DNase-seq) between vector control and PU.1 knockdown (shRNA 2) K562 over average signal found at each site. There are no significant DHS sites at P < 0.10 (n = 2 replicates). (c) MA plot of fold-change in chromatin accessibility between vector control and PU.1 knockdown (shRNA 2) K562 following 72-hour SAHA treatment over average signal found at each site. There are no significant DHS sites at P < 0.10 (n = 2 replicates). (d) Mean DNase-seq signal for vector control or PU.1 knockdown K562 in original SAHA-opened DHS sites that overlap a PU.1 ChIP-seq peak. RPM = reads per million mapped. (n = 2 replicates for vector control, n = 4 replicates for shRNA 1 and 2 combined). (e) Mean DNase-seq signal for vector control or PU.1 knockdown K562 in original SAHA-opened DHS sites that do not contain a PU.1 binding site. RPM = reads per million mapped. (n = 2 replicates for vector control, n = 4 replicates for shRNA 1 and 2 combined). [file 13072_2016_65_MOESM8_ESM.pdf]

# Supplementary Figure 6

**A**

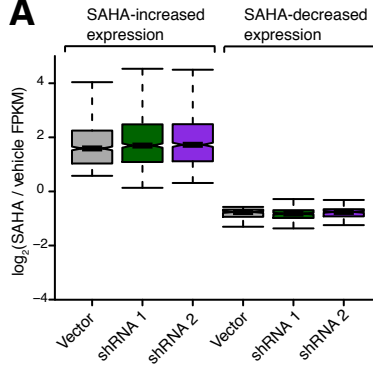

**B**

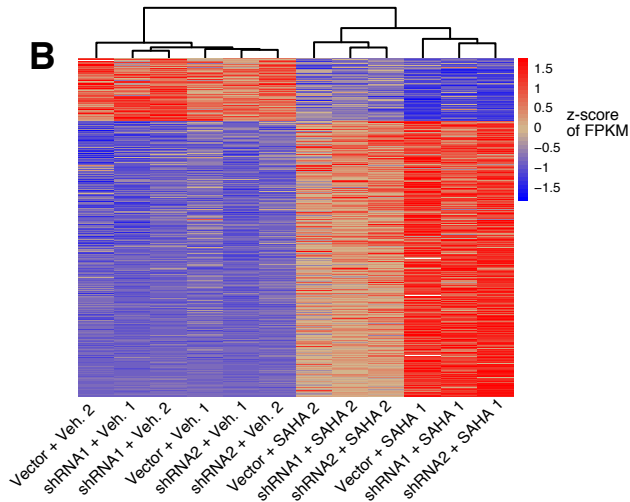

Supplement: Supplementary file 10 — 10.1186/s13072-016-0065-5 PU.1 depletion fails to block HDACi-induced gene expression changes in K562. (a) Boxplots of fold-change in RNA-seq expression for SAHA treatment of vector control-, shRNA 1-, or shRNA 2-transfected cells (n = 2 replicates) for genes that either increase or decrease significantly in vector control K562 (FDR < 0.05). (b) Heatmap of individual replicate expression values (FPKM values scaled across each row) for genes significantly differential following SAHA treatment in vector control K562. Note the distinction between all vehicle treated and all SAHA-treated samples and lack of distinction between PU.1 knockdown and vector control samples. [file 13072_2016_65_MOESM10_ESM.pdf]

# Supplementary Fig. 7

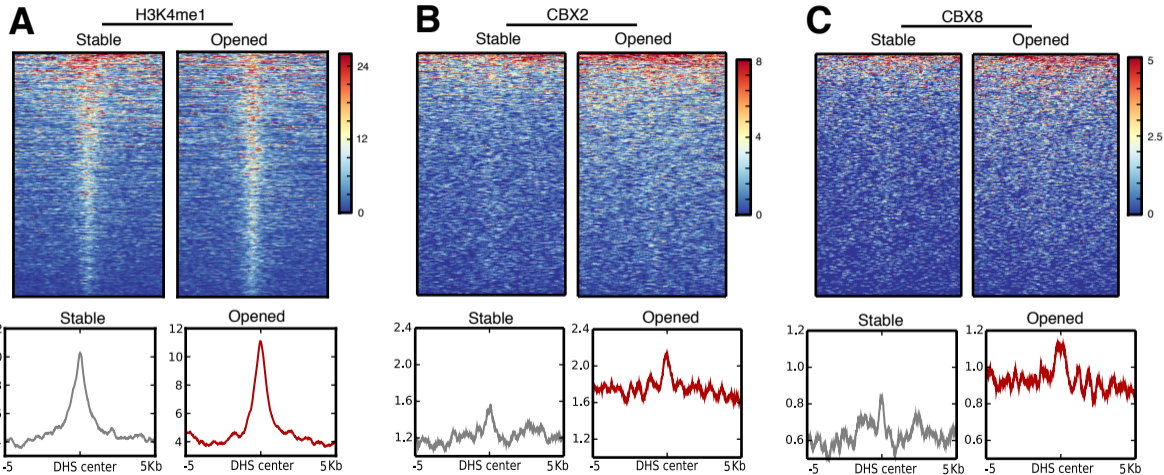

Supplement: Supplementary file 11 — 10.1186/s13072-016-0065-5 H3K4me1 enrichment and CBX2/8 binding at PU.1-bound DHS sites. Heatmaps of ChIP-seq signal present at each DHS site (DHS center +/− 5 kb) for the H3K4me1 mark (a), and chromobox proteins CBX2 (b) and CBX8 (c). Mean ChIP-seq signal present in the same regions plotted below. Note that H3K4me1 is similarly enriched in both opened and stable PU.1-bound DHS sites, while CBX2 and CBX8 display stronger signal in a subset of opened DHS sites and are therefore informative features for the random forest analysis presented in Fig. 6. [file 13072_2016_65_MOESM11_ESM.pdf]
