# Supplementary material for: HDAC inhibitors cause site-specific chromatin remodeling at PU.1-bound enhancers in K562 cells
Source: Epigenetics Chromatin. 2016 Apr 16;9:15. doi: 10.1186/s13072-016-0065-5 (PMC4833939; doi:10.1186/s13072-016-0065-5)
Supplement: Supplementary file 4 — 10.1186/s13072-016-0065-5 Motif enrichment in differential DHS sites. The top four enriched motifs (MEME-ChIP results) found in opened or closed DHS sites are listed with corresponding position weight matrix, expected (E) value, and top factor match found in the JASPAR or UniPROBE databases. [file 13072_2016_65_MOESM4_ESM.pdf]

Supplementary Figure 2

+ NaBut opened DHS

Motif:	E-value:	Top match:
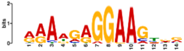	2.2×10^{-170}	SPI1 ("PU.1", MA0080.3)
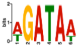	1.2×10^{-43}	GATA2 (MA0036.2)
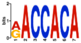	2.0×10^{-18}	RUNX1 (MA0002.2)
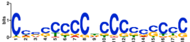	1.4×10^{-15}	SP2 (MA0516.1)

+ SAHA opened DHS

Motif:	E-value:	Top match:
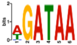	1.3×10^{-206}	GATA2 (MA0036.2)
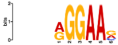	4.4×10^{-86}	SPI1 ("PU.1", MA0080.3)
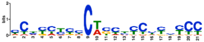	4.8×10^{-62}	ZNF263 (MA0528.1)
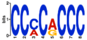	8.5×10^{-48}	KLF4 (MA0039.2)

+ NaBut closed DHS

Motif:	E-value:	Top match:
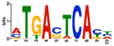	6.1×10^{-74}	FOSL1 (MA0477.1)
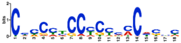	6.6×10^{-59}	ZNF263 (MA0528.1)
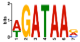	6.3×10^{-31}	GATA3 (MA0037.2)
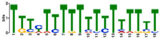	1.3×10^{-14}	MTF1 (UP00097_2)

+ SAHA closed DHS

Motif:	E-value:	Top match:
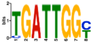	5.0×10^{-107}	NFYB (MA0502.1)
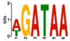	2.0×10^{-100}	GATA2 (MA0036.2)
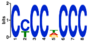	2.5×10^{-98}	KLF5 (MA0599.1)
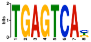	2.5×10^{-91}	FOS (MA0476.1)
